# Iatrogenic Aortocoronary Dissection During Right Coronary Artery Procedures: A Systematic Review of the Published Literature

**DOI:** 10.1016/j.jscai.2022.100443

**Published:** 2022-08-27

**Authors:** Erick Sanchez-Jimenez, Yaniv Levi, Ariel Roguin

**Affiliations:** a Cardiology Department, Hillel Yaffe Medical Center, Hadera, Israel; b Technion - Israel Institute of Technology, Israel

**Keywords:** aortocoronary dissection, catheter-induced aortic dissection, catheter-induced aortocoronary dissection, iatrogenic coronary dissection, stent

## Abstract

Iatrogenic aortocoronary dissection (IACD) occurs mainly during procedures involving the right coronary artery (RCA) and can result in disabilities, the need for urgent complex surgery, and even death. The risk factors for IACD are ill characterized, and the best management strategy is questionable; thus, there is a need to evaluate the characteristics, treatment options, and outcomes of patients with IACD of the RCA. We searched medical databases for publications on IACD of the RCA to present the characteristics of the procedures, management, and outcomes. We report 142 cases of IACD of the RCA, reported between 1973 and 2021. The mean age of the patients was 63.0 years, 81 (57%) were men, 75 (52.8%) presented with stable angina, and 29 (20.4%) had chronic total occlusion of the RCA. The most used catheter shapes were Judkins right (42%) and Amplatz left (25%), and most (56%) catheters were used during percutaneous coronary interventions. Guiding catheters were used in 38% (19/50) of diagnostic procedures when IACD occurred. A catheter size of ≤5F was used in only 3 cases. The catheter size was 6F in 22% of the cases, >6F in 23%, and not reported in 52%. A high-grade dissection (Dunning class III) occurred in 54% (77/142) of the cases. Stenting of the RCA ostium was performed in 88 (62%) of the cases, conservative treatment in 25 (18%), and surgery in 40 (28%) (aortic root repair [5%], coronary artery bypass grafting and aortic root repair [11%], and coronary artery bypass grafting alone [10%]). The mortality rate was 6.5% (5/77) among patients with class III dissection. Each patient should be considered independently. The most frequent intervention was to seal the dissection with a stent in the ostial RCA. However, in select cases published in the literature, a conservative approach was a feasible and successful option.

## Highlights

• We report on 142 procedures with RCA IACD reported between 1973 and 2021
• Mean age was 63 years, 57% were men, and 20.4% had RCA chronic total occlusion.
• The most used catheter shapes were JR (42%) and AL (25%).
• Treatment was stenting in 62%, conservative treatment in 18%, and surgery in 28%.
• The mortality rate was 5/77 (6.5%) among patients with class III dissection.

## Central Illustration

**Figure:**
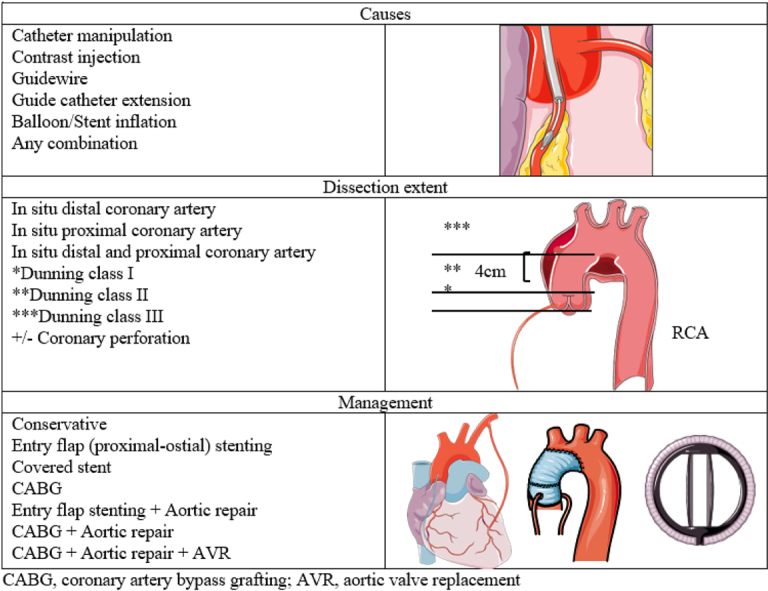

## Introduction

Iatrogenic aortocoronary dissection (IACD) during catheter manipulation at the time of diagnostic coronary angiography or percutaneous coronary intervention (PCI) is one of the most feared complications. The reported incidence of this complication is 0.02% to 0.15%.^1^, ^2^, ^3^ The dissection may spread to the ascending aorta and aortic valve, leading to a life-threatening condition, potential surgery, or even death. It can occur during urgent or elective procedures and in young individuals with normal coronary arteries or coronary arteries without significant stenosis.

Aortocoronary dissection is more common during engagement of the right coronary artery (RCA) ostium (>85% of cases) than that of the left coronary artery (LCA) (<15% of cases).^1^^,^^2^^,^^4^^,^^5^ There are structural and histologic differences between the proximal segment of the RCA and the LCA and between the right coronary sinus (RCS) of Valsalva and the left coronary sinus of Valsalva. The LCA is more resistant to traction and pressure than the RCA because of the size of the artery, angulation with the aorta, and histologic composition. Moreover, type I collagen fibers, known for their tensile strength, decrease in number in the ascending part of the sinuses, and the left portion of the sinotubular ridge has more smooth muscle cells and type I collagen than the right.^6^

The mechanism of dissection is not fully understood; however, it can range from catheter manipulation or contrast injection to wiring or ballooning.^7^, ^8^, ^9^ Dunning et al^3^ proposed an angiographic classification system: class I is defined as focal dissection restricted to the coronary cusp (aortic root), class II extends to the ascending aorta up to 40 mm, and class III extends to >40 mm from the ascending aorta (Central Illustration). To date, knowledge regarding the incidence of IACD and patients’ outcome based on treatment approach is lacking.^1^, ^2^, ^3^, ^4^, ^5^, ^6^, ^7^, ^8^, ^9^, ^10^

**Central Illustration:**
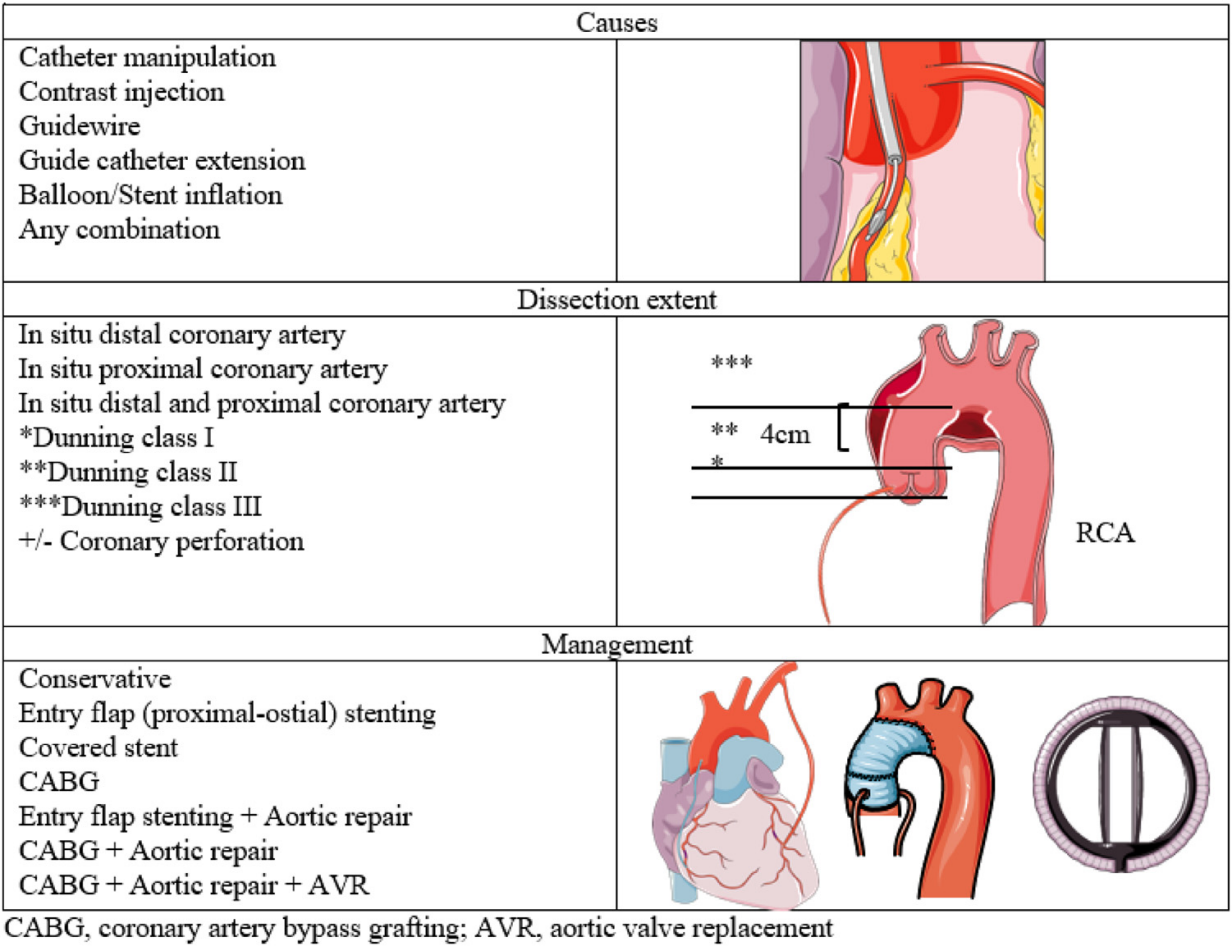
Aortocoronary dissection: mechanisms, types and management. AVR, aortic valve replacement; CABG, coronary artery bypass grafting.

In this report, we summarize what is published on IACD, with focus on right-sided IACD, which is more frequent. We present the characteristics of the procedures that resulted in IACD of the RCA as well as the management and outcomes of cases published in the literature to date.

## Methods

### Literature review strategy

A systematic review of the literature was conducted using PubMed and Google Scholar to identify cases of catheter-induced, right-sided IACD using the following keywords: “aortocoronary dissection,” “catheter-induced aortocoronary dissection,” and “catheter induced aortic dissection and iatrogenic coronary dissection.” Publications in English, Spanish, Italian, and German were included. From inception to May 2022, 1107 articles were identified (PubMed, 798; and Google Scholar, 309). Of these, 462 were duplicates; 461 were excluded because they dealt with different topics, such as spontaneous coronary artery dissection, contained (in situ) coronary artery dissection, and aortocoronary dissection caused by cardiac surgery procedures; 42 were about left main coronary artery dissection that extended to the aorta; 11 were literature reviews; and 39 had insufficient data. An additional 4 references were found using a manual search of the retrieved references. A total of 96 studies reporting 142 patients with right aortocoronary dissection met the inclusion criteria and were selected for complete analysis (Supplemental Table S1). 1, 2, 3, 4, 5^,^^10^, ^11^, ^12^, ^13^, ^14^, ^15^, ^16^, ^17^, ^18^, ^19^, ^20^, ^21^, ^22^, ^23^, ^24^, ^25^, ^26^, ^27^, ^28^, ^29^, ^30^, ^9^, ^31^, ^32^, ^33^, ^34^, ^35^, ^36^, ^37^, ^38^, ^39^, ^40^, ^41^, ^42^, ^43^, ^44^, ^45^, ^46^, ^47^, ^48^, ^49^, ^50^, ^51^, ^52^, ^53^, ^54^, ^55^, ^56^, ^57^, ^58^, ^59^, ^60^, ^61^, ^62^, ^63^, ^64^, ^65^, ^66^, ^67^, ^68^, ^69^, ^70^, ^71^, ^72^, ^73^, ^74^, ^75^, ^100^, ^76^, ^77^, ^78^, ^79^, ^80^, ^81^, ^82^, ^83^, ^84^, ^85^, ^86^, ^87^, ^88^, ^89^, ^90^, ^91^, ^92^, ^93^, ^94^, ^95^, ^96^, ^97^, ^98^, ^99^

### Statistical analysis

Descriptive statistics were used to report the clinical characteristics, angiographic details, management, follow-up, and outcomes among patients with right-sided IACD. Continuous variables are presented as means, and categorical variables are expressed as percentages, as appropriate.

## Results

We present the characteristics of 142 procedures that resulted in right-sided IACD, reported between 1973 and 2021 (Table 1). The mean age of the patients was 63.0 ± 10.2 years, 57% (81/142) of the patients were men, 52.8% (75/142) of the patients presented with stable angina, and 20.4% (29/142) of the patients had chronic total occlusion of the RCA (Supplemental Table S1). The most commonly used catheter shapes were Judkins right (JR) (42.0% [59/142]) and Amplatz left (AL) (25.4% [36/142]) (Table 2). Right-sided IACD occurred in 80 (56.3%) cases during PCI and 50 (35.2%) cases during diagnostic procedures. When IACD occurred during a diagnostic procedure, in 38% (19/50) of cases, a guiding catheter (GC) was used. The catheter size was ≤5F in only 3 (2.11%) cases, 6F in 32 (22.5%), 7F in 16 (11.3%), 8F in 15 (10.6%), and 9F in 2 (1.4%). The catheter size was unknown in 52.1% (74/142) of cases. Only 36 of the 142 cases also described the follow-up time, with a mean of 11.2 months per patient.

**Table 1:** Clinical and periprocedural characteristics of the cases analyzed with right iatrogenic aortocoronary dissection.

Case characteristics	Overall (N = 142)
Age, y	63.0
Male/female	81 (57.0)/59(41.5)
Diagnosis	
Stable angina	75 (52.8)
Unstable angina	21 (14.8)
NSTEMI	31 (21.8)
STEMI	11 (7.7)
Unknown	4 (2.8)
Chronic total occlusion cases	29 (20.4)
Dunning classification	
I	42 (29.6)
II	23 (16.2)
III	77 (54.2)
Diagnostic and follow-up tools beyond fluoroscopy	
CT	54 (38.0)
TEE	14 (9.9)
TTE	6 (4.2)
Nuclear scan	6 (4.2)
MRI	3 (2.1)
Only fluoroscopy	2 (1.4)
TEE + CT	2 (1.4)
TEE + MRI	1 (0.7)
Unknown	54 (38.0)
Management	
Conservative	25 (17.6)
Stenting	86 (60.6)
Stenting + aortic repair	3 (2.1)
Stenting + aortic repair + CABG	5 (3.5)
Stenting + AVR	1 (0.7)
CABG	15 (10.6)
CABG + aortic repair	7 (4.9)

Values are mean or n (%). AVR, aortic valve replacement; CABG, coronary artery bypass grafting; CT, computed tomography; MRI, magnetic resonance imaging; NSTEMI, non-ST-elevation myocardial infarction; STEMI, ST-elevation myocardial infarction; TEE, transesophageal echocardiogram; TTE, transthoracic echocardiogram.

**Table 2:** Coronary catheter types and procedure modality for patients with right iatrogenic aortocoronary dissection.

Catheter	Total cases, n (%)	Diagnostic, n	GC before PCI, n	GC during PCI, n	Unknown, n
JR	59 (41.5)	14	7	32	6
AL	36 (25.4)	3	2	28	3
AR	3 (2.1)		2	1	
HS	2 (1.4)		1	1	
Kimny	2 (1.4)		1	1	
Tiger	1 (0.7)	1			
JL	1 (0.7)			1	
Williams	1 (0.7)	1			
El-Gamal	1 (0.7)	1			
J Vector X	1 (0.7)		1		
Zuma	1 (0.7)			1	
Ikari L	1 (0.7)			1	
SAL1	1 (0.7)		1		
GC during diagnostic procedure	4 (2.8)		4		
GC during PCI	14 (9.9)			14	
DC or GC during diagnostic procedure	11 (7.7)	11			
Unknown	3 (2.1)				3
Total, n	142	31	19	80	12

AL, Amplatz left; AR, Amplatz right; DC, diagnostic catheter; GC, guiding catheter; HS, hockey stick; JL, Judkins left; JR, Judkins right; PCI, percutaneous coronary intervention; SAL1, short Amplatz left (Medtronic shape).

Three cases had anomalous origin of the RCA ostium from the left coronary sinus; 1 of them was treated with stenting of the RCA and left main coronary artery because of extension of the local dissection. One patient was treated with rotational atherectomy, and another had RCA perforation. One case was complicated by massive aortic regurgitation and was treated with aortic valve replacement.

In most cases, after the occurrence of IACD, an intervention was performed, and only 17.6% (25/142) of the patients were treated conservatively. Stenting of the RCA ostium was performed in 86 (60.6%) cases; stenting was followed by surgical aortic root repair in 9 (6.3%) cases, coronary artery bypass grafting (CABG) and aortic root repair in 7 (4.9%) cases, and CABG alone in 15 (10.6%) cases. Of the patients treated with stents, 5 were managed with covered stents, 1 because of RCA perforation and the other because of lack of sealing of the entry flap after placement of a regular drug-eluting stent.

The majority of patients with Dunning class I (42 cases) were managed with either stents or conservatively, and only 2 of them underwent aortic repair, with no fatalities. Among patients with Dunning class II (23 cases), 4 directly underwent CABG and only 1 underwent aortic repair. Only 1 patient died in this group. In the class III group (77 cases), 31 underwent some type of open-heart surgery; of them, 12 patients underwent aortic repair. We found that 6.5% (5/77) of patients in the class III group died, reflecting the relatively poor outcomes in this group.

Among the 142 patients, we found a total of 6 (4.2%) deaths, all of which were in-hospital after the procedure (Table 3). Of the 6 deceased patients, 5 had high-grade Dunning III IACD; 4 were women; and 2 were treated conservatively, 1 with stenting, 2 with initial stenting, followed by aortic repair and CABG, and 1 directly with aortic repair and CABG.

**Table 3:** Clinical and periprocedural characteristics of deceased cases with right iatrogenic aortocoronary dissection.

Case characteristics	Overall (N = 6)
Age, y	63.2
Female	4 (66.7)
Diagnosis	
Stable angina, chronic total occlusion	1 (16.7)
Unstable angina	1 (16.7)
NSTEMI	2 (33.3)
STEMI	1 (16.7)
Unknown	1 (16.7)
Dunning classification	
I	0 (0)
II	1 (16.7)
III	5 (83.3)
Procedure	
Diagnostic	3 (50)
During percutaneous coronary intervention	3 (50)
Management	
Conservative	2 (33.3)
Stenting	1 (16.7)
Stenting + aortic repair + CABG	2 (33.3)
CABG + aortic repair	1 (16.7)

Values are mean or n (%). CABG, coronary artery bypass grafting; CTO, chronic total occlusion; NSTEMI, non-ST-elevation myocardial infarction; STEMI, ST-elevation myocardial infarction.

## Discussion

Our report, which comprises the largest series of case reports on IACD gathered together, highlights few factors regarding the causes of and treatment options for right-sided IACD. The vast majority of IACDs occurred during PCIs, with a high percentage occurring in patients with chronic total occlusion. The reason for IACD can be catheter manipulation or PCI related. Based on the assessment of catheter size, we observed that the majority of the catheters were ≥6F; however, the size of many (52%) was unknown.

Right-sided IACD can occur during catheter engagement in diagnostic procedures or during any step of PCIs. A dissection usually begins in the proximal portion or ostium of the RCA and propagates to the RCS and eventually to the ascending aorta but can also be contained or propagated distally into the coronary vessel wall. Maintaining coaxial alignment of the catheter is crucial and can decrease the risk of dissection, especially when more aggressive equipment is used during PCIs such as atheroablative techniques.

**Figure 1:**
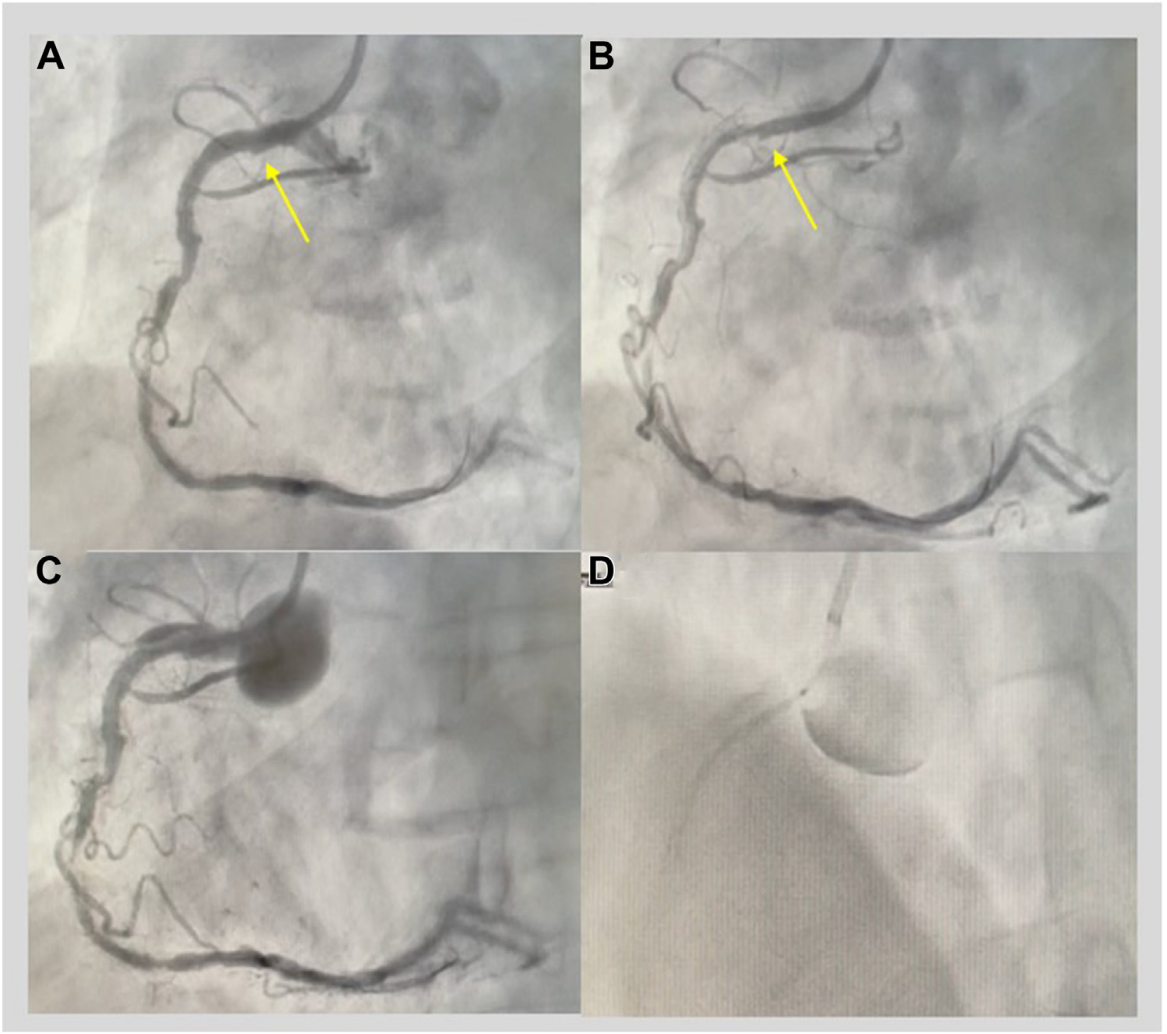
**Right coronary artery (RCA) iatrogenic aortocoronary dissection**. A 66 year-old man with ST-elevation myocardial infarction. (**A**) RCA angiography using the Amplatz left 0.75 guiding catheter. The arrow points to the tip of the catheter pulling on the inferior wall of the proximal arterial segment and deep cannulation, best seen in (**B**), just after contrast washout. (**C**) The next contrast injection with a proximal D-type RCA dissection with retrograde extension to the right coronary sinus, in which the contrast is contained. (**D**) Placement of a drug-eluting stent protruding from the coronary ostium with the intention of sealing the flap and preventing extension of the dissection into the ascending aorta.

The diagnosis of IACD is made immediately using angiography, and prompt identification is crucial in order to avoid further contrast injections and further spread of the dissection (Figure 1). A dissection appears as an intimal tear with a filling defect within the coronary lumen or as an extraluminal cap with persistent contrast, sometimes with total occlusion of the coronary artery lumen. Some patients may be clinically more stable and present without chest pain or electrocardiographic changes; however, others may present more dramatically with acute chest pain, significant electrocardiographic changes, ventricular arrhythmias, and hypotension.

There are neither well-controlled studies nor clear guidelines on when IACD occurs. Further contrast injections may extend the dissection and should be avoided. Intravascular imaging, especially intravascular ultrasound (IVUS), is a useful tool for finding the dissection entry point; can guide the stent repair procedure, ensuring complete coverage of the ostium and flap; can help choose the best size for the stent; and can guide PCIs. Moreover, IVUS may be useful for determining the progress of retrograde dissection, involvement of the RCS, degree of dissection and, therefore, help decide whether or not to place a stent.^21^^,^^22^^,^^30^ Optical coherence tomography involves contrast injections and should be avoided.

Among patients who developed IACD during a diagnostic procedure, the rate of the use of a GC was high. Assuming that the most common catheter used in this scenario is diagnostic, this hints that the use of GCs in diagnostic cases may increase the risk of IACD. JR4 and AL1 were the 2 catheter shapes that were most used during this complication; however, we did not know the total number and shapes of the GCs used and, thus, could not calculate the rate of complications for each device or for a particular manufacturer.

When IACD occurs, the treatment options are conservative: stenting of the RCA ostium or surgical aortic root repair, with or without the need for surgical revascularization. Based on an assessment of the patients’ outcomes according to IACD severity and treatment approach, we found that the majority of patients with low-grade IACD (class I Dunning calcification, 42 cases) were managed either with stents or conservatively; only 4 (9.5%) underwent open-heart surgery, 2 directly underwent CABG, and 2 underwent aortic repair after stenting of the ostial RCA, with no fatalities. In the class II group (23 cases), 5 (21.7%) patients underwent cardiac surgery; among them, 4 directly underwent CABG and 1 underwent aortic repair after stenting. In this group, only 1 patient (4%) died. The majority of the patients in our study—54% (77/142)—presented with class III severity of IACD, of whom 40% (31/77) underwent open-heart surgery, and 10.6% (15/142) also underwent aortic repair. However, there may have been a significant publication bias because cases with minor dissection might have been less likely to be reported (classes I and II). Thus, the surgical rate bailout may be less. Among patients with class III dissection, the mortality rate was 6.5%, reflecting the poor outcomes in this last group. Of the 6 deaths, 4 (66.6%) were in women. Worse prognoses in women have been suggested by previous reports.^101^

According to the data above, it is confirmative that the vast majority of IACD of the RCA, especially with the class III Dunning classification, should be treated with stenting of the RCA ostium containing the entry flap and sealing the opening. However, there are no high-quality studies dealing with this topic. There is no evidence-based information that this management prevents further extension of the dissection to the aortic root or ascending aorta beyond the RCS; yet, we probably will not see such a trial. This is why studies such as the present one, which summarizes what was done and what the prognosis was, may suggest the best course of action to consider in such rare but potentially catastrophic scenarios.

It is noteworthy that 25 (17.6%) cases were managed conservatively, even without stenting, and some cases had a follow-up of 6 to 36 months, without any major adverse cardiovascular events. In this group, 2 deaths were reported. Of note, some of the cases reported are also from a period when stenting was not present or had just begun and were less frequently used.

After IACD occurs in a catheterization laboratory, using either conservative management or ostial RCA PCIs, close attention must be given and follow-up carried out in order to determine whether any other strategy, such as CABG, aortic root repair, and aortic valve replacement, must be applied. The information obtained in this study regarding follow-up was not optimal; however, we can say that patients evaluated using only fluoroscopy, echocardiograms, transesophageal echocardiograms, and nuclear scans were from older publications, mostly prior to 2008.

The classification system by Dunning et al^3^ was developed in an era before the widespread availability and use of intravascular imaging techniques, such as IVUS, and noninvasive imaging using computed tomography angiography. In the most contemporary cases, aortic computed tomography was used, which is the gold standard to date; if a conservative approach was chosen, repeating computer tomography in 4 to 6 weeks may have been valuable to ensure healing of the dissection.^102^ Transthoracic echocardiograms are essential for evaluating the aortic valve and other structures.

We must acknowledge several limitations. First, there is a publication bias because many patients who did not survive were not reported. Naturally, one aims to publish mainly successful reports. Second, we could not calculate the incidence of IACD because we did not know the total number of procedures. Third, we did not know why a particular shape of catheter was chosen; this may have been related to more complex anatomy. Fourth, in recent years, we have seen a change from a femoral approach to a radial approach; however, most of the cases did not mention the access route, and no conclusion could be reached regarding IACD and access. The information obtained in this study regarding follow-up was not optimal; only 36 cases described the follow-up time beyond hospital stay.

## Conclusion

In the present report, comprising the largest series of case reports on IACD collected so far, we found that iatrogenic dissection of the RCA and its extension to the aortic root and eventually to the ascending aorta is a rare but potential complication that every interventional cardiologist must take into account while performing both diagnostic procedures and PCIs, especially in patients with chronic total occlusions. Although virtually all catheters can induce such a complication, the most common are GCs with a size of ≥6F, specifically JR4 and AL1 GCs. The mechanism and causes can vary with the catheter itself, contrast injection, guide wire injury, guide extensions, balloons, and stents. However, the exact etiology remains an intriguing concept and may be associated with some predisposition of the tissue to traction.

The high-grade Dunning classification system shows the worst outcome in patients when an aortocoronary dissection extends to the ascending aorta. Yet, in spite of the inherited bias of this series of publications, most patients with this complication can be treated successfully.

In summary, the conservative approach to right-sided IACD is a feasible option in selected patients with stable presentation with close follow-up; however, stent implantation to seal the flap in the ostial RCA is the most common management strategy and has the highest success rate, reserving CABG and aortic repair for unstable patients with confirmatory progression of the dissection to the ascending aorta and beyond, as detected using computed tomography aortography.
