# Supplementary material for: Iatrogenic Aortocoronary Dissection During Right Coronary Artery Procedures: A Systematic Review of the Published Literature
Source: J Soc Cardiovasc Angiogr Interv. 2022 Aug 27;1(6):100443. doi: 10.1016/j.jscai.2022.100443 (PMC11308116; doi:10.1016/j.jscai.2022.100443)
Supplement: Supplementary Data [file mmc1.docx]

Supplementary Table 1. Published cases of right aortocoronary dissection during catheterization.

| **Author / year / ref.** | **#** | **Age** | **M/F** | **Diagnosis** | **Dunning class** | **Cath** | **Dx/PCI, lesion** | **Treatment** | **Diag. Tool** | **FU** |
| --- | --- | --- | --- | --- | --- | --- | --- | --- | --- | --- |
| Hung J, 2021. (10) | 1 | 78 | M | SA | III | AL 0.75 GC | Diagnostic | Stenting | CT |  |
| Goldstein JA, 2003. (12) | 1 | 62 | M | STEMI | II | JR4 GC | During PCI | Stenting | CT |  |
| Carstensen S, 2008. (11) | 1 | 64 | F | SA  R-CTO | III | JR4 GC | During PCI | Stenting | CT |  |
|  | 2 | 57 | M | SA | I | AL1 GC | During PCI | Stenting | CT |  |
|  | 3 | 56 | F | SA | III | JR4 GC | During PCI | Stenting | CT |  |
| Gurdal A, 2018. (13) | 1 | 64 | F | SA |  | JR4 DC | Diagnostic | Stenting | CT |  |
| Artemiou P, 2016. (14) | 1 | 57 | F | NSTEMI | II |  | Diagnostic | CABG |  |  |
| Santos M, 2011. (15) | 1 | 56 | M | SA  R-CTO | III | AL2 GC | During PCI | Stenting | CT |  |
| Shuto T, 2017. (16) | 1 | 72 | F | SA  R-CTO | III | AL1 GC | During PCI | Stenting | CT |  |
| Cereda AF, 2020. (17) | 1 | 84 | M | SA | III | JR4 GC | During PCI | Stenting |  |  |
| Lee SI, 2018. (18) | 1 | 55 | F | UA | II | GC | Diagnostic | Conservative | CT |  |
| Liao MT, 2012. (19) | 1 | 53 | M | SA  R-CTO | III | AL1 GC | During PCI (Wiring) | Stenting | CT |  |
| Tang L, 2017. (20) | 1 | 62 | F | UA | III | Williams | Diagnostic | Stenting | CT |  |
|  | 2 | 60 | M | SA | II | 5F JR 4 GC | During PCI | Stenting | CT |  |
|  | 3 | 63 | F | SA | I | 6F JL 3.5 GC | During PCI | Stenting | Angiography |  |
|  | 4 | 52 | F | UA | III | 6F AL1 GC | During PCI | Stenting | CT |  |
| Oda H, 2004. (21) | 1 | 70 | M | UA  R-CTO | III | 7F AL1 | During PCI | Stenting | CT |  |
| Abdou SM, 2011. (22) | 1 | 58 | M | UA  R-CTO | III | 6F Ikari Left GC | During PCI | Stenting | CT |  |
| Bansal V, 2020 (23) | 1 | 63 | F | SA | III | JR4 DC | Diagnostic | Aortic R. + CABG |  |  |
| Dahdouh Z, 2012. (24) | 1 | 70 | F | SA | III | AL 0.75 GC | During PCI | Stenting + AVR |  |  |
| Chong S, 2022. (25) | 1 | 61 | M | STEMI (RCA from LCS) | III  LM | 6F SAL1 GC | Diagnostic | Stenting RCA and LM |  |  |
| Kassimis G, 2018. (26) | 1 | 52 | M | STEMI | III | 6F JR4 GC | During PCI | Stenting | CT | 3d |
|  | 2 | 55 | F | NSTEMI | I | 6F JR4 GC | During PCI | Stenting | CT | 2d |
| Baumann S, 2014. (27) | 1 | 71 |  | SA  R-CTO | III | Hockey Stick GC | During PCI | Stenting | CT |  |
| Kim JY 2005. (28) | 1 | 67 | M | SA | III | 6F Kimny GC | Diagnostic | Stenting | CT |  |
| Hunt I, 2006. (29) | 1 | 62 | F | SA | III | GC | During PCI | CABG |  |  |
|  | 2 | 73 | M | SA  R-CTO | III | GC | During PCI (Wiring) | CABG |  |  |
| Sakakura K, 2013. (30) | 1 | 79 | M | SA | III | GC | During PCI | Stenting | CT |  |
| Al Salti Al Krad H, 2014. (31) | 1 | 65 | F | SA  R-CTO | I | AL1 | During PCI | Conservative |  |  |
| Dandale R, 2017. (32) | 1 | 83 | M | SA | III | JR4 GC | During PCI (Rota) | Stenting | CT |  |
| Fang h, 2011. (33) | 1 | 66 | M | SA | III | AL2 | During PCI | Stenting | CT |  |
| Saito T, 2011. (34) | 1 | 67 | M | SA  (RCA from LCS) | II | 7F AL2 | During PCI | Stenting | CT |  |
| Sarkis A, 2007. (35) | 1 | 45 | F | UA | III | JR DC | Diagnostic | CABG |  |  |
| Seifein HB,1996. (36) | 1 | 48 | M | SA | II | 8F JR4 | During PCI (balloon) | CABG |  |  |
| Bae JH, 1998. (37) | 1 | 55 | M | NSTEMI | III | 6F JR4 GC | Diagnostic | Stenting |  |  |
| Baumann S, 2017. (38) | 1 | 69 mean | M | SA  R-CTO | III | Hockey Stick GC | Diagnostic | Stenting | CT |  |
|  | 2 | 69 mean | M | SA | III | AL1 | Diagnostic | Stenting | CT |  |
|  | 3 | 69 mean | M | SA | I | GC | During PCI (GL) | Stenting | CT |  |
|  | 4 | 69 mean | M | NSTEMI R-CTO | III | AL1 | During PCI (GL) | Conservative |  |  |
|  | 5 | 69 mean | F | SA | II | AL1 | Diagnostic | Conservative | CT |  |
|  | 6 | 69 mean | F | UA | III | AR2 GC | Diagnostic | Stenting | CT |  |
|  | 7 | 69 mean | F | SA  R-CTO | I | AL1 | During PCI (GL) | Stenting | CT |  |
| Okamoto R, 2000. (39) | 1 | 67 | M | SA | III | JR4 | During PCI | Stenting | CMR |  |
| Abu-Ful A, 2003. (40) | 1 | 74 | M | SA | III | 7F Zuma GC | During PCI | Covered stent |  |  |
| Neo W, (41) 2009. | 1 | 54 | M | SA | III | 4F JR DC | Diagnostic | CABG |  |  |
| Rangel-Abundis A, 2005. (42) | 1 | 54 | F | SA | III | 8F JR4 DC | Diagnostic | Stent + Aortic R. + CABG | CT |  |
| Nasrin S, 2017. (43) | 1 | 60 | M | SA | III | 6F JR 3.5 GC | During PCI | Covered stent (RCA perforation) |  |  |
| Geraci A, 1973. (44) | 1 | 65 | F | UA | III |  | Diagnostic | Conservative |  |  |
| Rao G, 1975. (45) | 1 | 46 | F | SA | III |  | Diagnostic | CABG |  |  |
| Darwarzah A, 2008. (46) | 1 | 46 | M | SA | III | 6F JR DC | Diagnostic | CABG |  |  |
| Tanasie C, 2011. (47) | 1 | 80 | F | NSTEMI | III |  | Diagnostic | Stenting |  |  |
|  | 2 | 58 | M | UA | II |  | Diagnostic | Conservative |  |  |
|  | 3 | 79 | M | NSTEMI | II |  | Diagnostic | Stenting |  |  |
|  | 4 | 56 | M | SA | I | GC | During PCI | Stenting |  |  |
|  | 5 | 74 | M | NSTEMI | I | GC | During PCI | Stenting |  |  |
|  | 6 | 70 | F | SA | III |  | Diagnostic | Stenting |  |  |
|  | 7 | 73 | M | UA | III |  | Diagnostic | CABG |  |  |
| Antoniou A, 2017. (48) | 1 | 80 | F | SA | III | AL1 GC | During PCI | Stenting | CT |  |
| Fiddler M, 2015. (49) | 1 | 65 | F | NSTEMI R-CTO | III | AL1 GC | During PCI | Stenting | CT |  |
| Mamas MA, 2008. (50) | 1 | 72 | F | SA  R-CTO | III | AL1 GC | During PCI (Wiring) | Conservative | CT |  |
| Sohrabi B, 2007. (51) | 1 | 50 | M | UA | III | 6F JR4 DC (Medtronic*)* | Diagnostic | Stenting | TEE |  |
| Park IW, 2008. (52) | 1 | 68 | F | SA | III | 7F JR4 | Diagnostic | Stenting | CT |  |
| Roumy A, 2016. (53) | 1 | 74 | F | SA | I | GC | During PCI | Conservative | CT |  |
| Dunning DW, 2000.  (3) | 1 | 77 | F | SA | I | 9F JR4 | During PCI | Stenting |  |  |
|  | 2 | 57 | F | NSTEMI | II | 8F AL1 GC | During PCI | Stent + Aortic R. + CABG | TEE |  |
|  | 3 | 61 | F | NSTEMI | III | 7F JR4 | During PCI | Stent + Aortic R. + CABG |  | IH death |
|  | 4 | 61 | M | SA | I | 8F AL1 | During PCI (Rotablation) | Stenting | CT |  |
|  | 5 | 42 | M | NSTEMI | II | 8F JR4 GC | Diagnostic | Stenting |  |  |
|  | 6 | 63 | M | NSTEMI | III | 9F AL2 | Diagnostic | Stent + Aortic R. + CABG |  | IH death |
|  | 7 | 55 | M | SA | II | 8F AL1 | During PCI | Stenting |  |  |
|  | 8 | 50 | M | SA | I | 6F JR4 DC | Diagnostic | Stenting | TTE |  |
|  | 9 | 75 | M | SA | I | 8F JR4 GC | During PCI | Stenting | TTE |  |
| Carter AJ, 1993. (54) | 1 | 70 | M | SA | III | 8F AL2 GC | During PCI | Conservative | CMR | 36m |
|  | 2 | 57 | F | SA | III | 8F JR4 GC | During PCI | Conservative | CMR | 36m |
|  | 3 | 48 | M | SA | III | 6F JR4 DC | Diagnostic | Conservative | TTE | 36m |
| Boukhris M, 2015. (9) | 1 | 62 | M | SA  R-CTO | II | JR4 GC | During PCI | Stenting | CT | 52m |
|  | 2 | 66 | M | SA  R-CTO | II | JR4 GC | During PCI | Stenting | TEE | 48m |
|  | 3 | 65 | M | SA  R-CTO | II | AL1 GC | During PCI | Covered stent | CT | 36m |
|  | 4 | 59 | M | SA  R-CTO | I | JR4 GC | During PCI | Stenting | CT | 32m |
|  | 5 | 53 | M | SA  R-CTO | I | JR4 GC | During PCI | Stenting | CT | 22m |
|  | 6 | 71 | F | SA  R-CTO | II | JR4 GC | During PCI | Stenting | CT | IH death |
|  | 7 | 69 | M | SA  R-CTO | II | AR1 GC | During PCI | Covered stent | CT | 3m |
| Koza Y, 2018. (55) | 1 | 74 | M | NSTEMI | II | JR4 DC | Diagnostic | CABG |  |  |
| Bekler A, 2014. (56) | 1 | 70 | F | NSTEMI | III | 6F JR4 GC (Medtronic) | Diagnostic | Stenting | CT |  |
| Nikolsky E, 2003. (57) | 1 | 40 | M | NSTEMI | I | 7F JR4 DC | During PCI | Conservative |  | 24m |
| Doyle B, 2004. (58) | 1 | 64 | F | SA | III | 8F AL1 GC (Boston) | During PCI | Conservative | TEE | 6m |
| St.Goar FG, 1991. (59) | 1 | 78 | M | NSTEMI | I | 6F JR DC | Diagnostic | Stenting |  | 1m |
| Gur M, 2006. (60) | 1 | 50 | M | UA | I | 7F JR DC (Medtronic) | Diagnostic | CABG |  |  |
| Yip HK, 2001. (2) | 1 | 68 | M | NSTEMI | I | 7F JR4 | During PCI (Balloon) | Stenting | Nuclear scan | 6m |
|  | 2 | 80 | M | NSTEMI | I | 7F AL2 | During PCI | Stenting | Nuclear scan | 6m |
|  | 3 | 73 | M | UA | III | 7F JR4 | During PCI (Balloon) | Conservative |  | IH death |
|  | 4 | 62 | F | NSTEMI | I | 7F JR4 | During PCI (Balloon) | Stenting | Nuclear scan | 6m |
|  | 6 | 63 | M | UA | I | 6F AL2 | During PCI (GC) | Stenting | Nuclear scan | 6m |
|  | 7 | 66 | M | UA | I | 6F JR4 | During PCI (Balloon) | Stenting | Nuclear scan | 6m |
| Kwan T, 1995. (61) | 1 | 45 | F | UA | I | 7F JR DC | Diagnostic | Conservative | Nuclear scan | 3m |
| Perez-Castellano N, 1998. (1) | 1 | 67 | F |  | I | GC | During PCI (balloon) | Stenting | TEE |  |
|  | 2 | 52 | M |  | I | GC | Diagnostic | Conservative |  |  |
|  | 3 | 56 | F |  | III |  | Diagnostic | Aortic R. + CABG |  | IH death |
|  | 4 | 64 | M |  | I | GC | During PCI (balloon) | Stenting |  |  |
| Alfonso F, 1997. (62) | 1 | 62 | M | NSTEMI | II | GC | During PCI | Stenting | TEE | 12m |
|  | 2 | 70 | M | UA  R-CTO | III | GC | During PCI (wiring) | CABG |  |  |
| Moles V, 1992. (4) | 1 | 61 | M | UA  R-CTO | III | 8F AL2 GC | During PCI (wiring) | Aortic R. + CABG | TEE | 6d |
| Varma V, 1992. (63) | 1 | 55 | F | STEMI | III | GC | Diagnostic | Conservative | TEE | IH death |
| Pande A, 1996. (64) | 1 | 55 | W | NSTEMI R-CTO | III | 8F JR GC | During PCI (balloon) | CABG | TTE |  |
| Mauser M, 1999. (65) | 1 | 75 | F | SA  R-CTO | I | 7F JR GC (Medtronic) | During PCI (GC) | CABG | TTE |  |
| Wagdi P, 1999. (66) | 1 | 71 |  | SA | II | JR4 GC | Diagnostic | Stenting | CT | 6m |
| Brown RA, 2009. (67) | 1 | 57 | M | STEMI | III | 6F AL1 GC (Cordis) | During PCI | Covered stent | CT | 4d |
| Sekiguchi M, 2009. (68) | 1 | 81 | M | NSTEMI  (RCA from LCS) | I | AL0.75 GC (Boston Scientific) | During PCI | Stenting | CT | 21d |
| Leclercq F, 2000. (69) | 1 | 65 | F | SA | II | 7F J Vector X GC  (Medtronic) | Diagnostic | CABG | TEE |  |
| Tam DY, 2016. (70) | 1 | 50 | F | STEMI | III | GC | During PCI (stent) | Conservative | CTx4 | 4m |
| Kagoshima M, 2002. (71) | 1 | 75 | M | SA | II | 6F Kimny GC (Schneider) | During PCI | Stenting | CT |  |
| Kostov J, 2003. (72) | 1 | 54 | M | SA  R-CTO | I | 8F AL GC (Cordis) | During PCI (wiring) | Conservative | Angiographic | 3m |
| Ahmed AA, 2001. (5) | 1 | 45 | M | SA | I | JR4 GC (cordis) | During PCI (balloon) | Stent + Aortic R. |  |  |
| Notaristefano S, 2005. (73) | 1 | 45 | M | STEMI (left)  R-CTO | III | 6F JR4 (Cordis) | During PCI | Stenting | CT | 6m |
| Shah P, 2016. (74) | 1 | 52 | M | STEMI | III | 6F JR4 GC | During PCI | Stenting | TEE | 4d |
| Sutton AG, 2000. (75) | 1 | 52 | M | SA | III | El-Gamal | Diagnostic | Aortic R. + CABG |  |  |
| Hung MJ, 2002. (76) | 1 | 64 | F | SA | I | 6F |  | Stenting |  |  |
|  | 2 | 51 | M | NSTEMI | I | 6F JR |  | Stenting |  |  |
| Maiello L, 2003. (77) | 1 | 48 | F | SA | III | 6F JR |  | Stenting | TEE |  |
| Masaki Y, 2005. (78) | 1 | 82 | F | NSTEMI | III | 6F AL |  | Conservative |  |  |
| Pohlel K, 2006. (79) | 1 | 62 | F | NSTEMI | I | JR4 GC (Cordis) | Diagnostic | Stenting | TEE + CT |  |
| Minicucci F, 2006. (80) | 1 | 60 | F | NSTEMI | I | 6F GC | During PCI (Balloon) | Stenting | TEE | 1m |
| Papadopoulos DP, 2006. (81) | 1 | 52 | M | NSTEMI | III | JR4 |  | Stenting |  |  |
| Colkesen AY, 2007. (82) | 1 | 79 | F | STEMI | III | 6F JR GC | During PCI (balloon) | Conservative | CT |  |
| Uyan C, 2008. (83) | 1 | 79 | F | SA | I |  |  | Conservative |  |  |
| Cohen R, 2008. (84) | 1 | 42 | M | STEMI | III | 6F AR2 GC | Diagnostic | Aortic R. + CABG |  |  |
| Hacibayramoglu M, 2008. (85) | 1 | 73 | F | SA | III | JR | During PCI (stenting) | Stent + Aortic R. |  |  |
| Ziakas AG, 2009. (86) | 1 | 54 | M | UA | III | GC | Diagnostic | Stent + Aortic R. | CT |  |
| Jarmoszewicz K, 2009. (87) | 1 | 70 | F | NSTEMI | 1 |  |  | Stent + Aortic R. + CABG |  |  |
| Wykrzykowska JJ, 2012. (88) | 1 | 61 | F | NSTEMI | III | AL2 |  | Conservative |  |  |
| Danzi GB, 2012. (89) | 1 | 73 | F | SA | III | 6F JR |  | Stenting |  |  |
| Burstow D, 2013. (90) | 1 | 53 | M | NSTEMI | I | 6F JR4 |  | Stenting | TEE | 3m |
| Alfonso F, 2004. (91) | 1 | 46 | M | UA  R-CTO | I | GC | During PCI (Balloon) | Stenting | TEE + MRI |  |
| Bryniarski L, 2008. (92) | 1 | 67 | M | SA  CTO | I | 6F JR (Medtronic) GC | Diagnostic | Conservative | TEE | 1 m |
| Chunlai S, 2012. (93) | 1 | 59 | F |  | III | 7F JR4 |  | Conservative | CT |  |
| Pentousis D, 2000. (94) | 1 | 63 | M | SA | III | 7F AL1 | During PCI | Stenting |  |  |
| Pai RK, 2005. (95) | 1 | 58 | M | SA | III |  |  | Aortic R. + CABG |  |  |
| Garg P, 2009. (96) | 1 | 83 | M | SA | III | 8F AL0.75 GC |  | Stenting |  |  |
| Salvatore S, 2018. (97) |  | 65 | F | STEMI |  | 6F JR4 GC | Diagnostic | Stenting | CT |  |
| Goel A, 2021. (98) | 1 | 82 | M | UA | II | AL2 GC | Diagnostic | Stenting | TTE | 5d |
| Damera SR, 2017. (99) | 1 | 59 | F | NSTEMI | III | 5F Tiger DC | Diagnostic | Stenting | CT | 1m |
| Yarlioglues M, 2010. (100) | 1 | 53 | F | SA | III |  | Diagnostic | Aortic R + CABG | CT |  |

NSTEMI, non-ST elevation myocardial infarction, STEMI, ST-elevation myocardial infarction; SA, stable angina, UA, unstable angina; HS, Hockey-Stick; K, Kimny; GL, guideliner; Aortic R, Aortic repair; AL, amplatzer left; JR, Judkins right; F, French; CT, computer tomography; CMR, cardiac magnetic resonance; d, days; m, months; PCI, percutaneous coronary intervention; GC, guiding catheter; DC, diagnostic catheter; R-CTO; CABG, coronary artery bypass grafting; AVR, aortic valve replacement; TEE, transesophageal echocardiogram; IH, in-hospital; TTE, Transthoracic echocardiogram.
